# Stress fluoroscopic evaluation of dynamic knee stability after medial open-wedge high tibial osteotomy: preoperative instability predicts postoperative stabilization

**DOI:** 10.1016/j.asmart.2026.10.001

**Published:** 2026-10-23

**Authors:** Yoshiki Okazaki, Takashi Maehara, Hiroaki Kadota, Kousaku Kubota, Naoya Chikaishi

**Affiliations:** a Department of Orthopaedic Surgery, Kagawa Prefectural Central Hospital, 1-2-1 Asahimachi, Takamatsu, Kagawa, 760-8557, Japan; b Department of Orthopaedic Surgery, Kagawa Rosai Hospital, 3-3-1 Joto-cho, Marugame, Kagawa, 763-8502, Japan

**Keywords:** Dynamic knee stability, High tibial osteotomy, Joint line convergence angle, Medial meniscus posterior root tear, Posterior tibial slope

## Abstract

**Purpose:**

Medial open-wedge high tibial osteotomy (OWHTO) is widely performed for medial compartment pathology associated with varus malalignment. Although OWHTO corrects static alignment, its influence on dynamic knee stability remains unclear. This study aimed to evaluate changes in coronal and sagittal dynamic stability after OWHTO using intraoperative stress fluoroscopic assessment.

**Methods:**

Thirty-eight knees in 35 patients undergoing OWHTO were retrospectively reviewed. Coronal stability was assessed using dynamic joint line convergence angle (dJLCA) and dynamic femorotibial angle (dFTA), and sagittal stability was evaluated using anterior tibial translation relative to Blumensaat's line (ATB). Alignment parameters included femorotibial angle (FTA), medial proximal tibial angle (MPTA), and posterior tibial slope (PTS). Clinical outcomes were assessed using Lysholm, IKDC, and KOOS scores up to 12 months postoperatively. Pre- and postoperative differences were analyzed using the Wilcoxon signed-rank test, and correlations were evaluated using Spearman's rank correlation.

**Results:**

All alignment and stability parameters significantly improved postoperatively (p < 0.001). dJLCA and ATB showed marked reductions, indicating decreased stress-induced coronal and sagittal instability. Clinical scores significantly improved at 12 months (p < 0.001). Greater preoperative instability was strongly correlated with greater postoperative stabilization (dJLCA ρ = 0.794; dFTA ρ = 0.765; ATB ρ = −0.765; PTS ρ = 0.530).

**Conclusions:**

OWHTO improves coronal and sagittal dynamic stability under stress conditions. These findings suggest that dynamic instability in varus knees is largely caused by malalignment and can be improved by osteotomy. Stress fluoroscopic assessment may aid in identifying correctable instability and guiding surgical planning.

**Level of evidence:**

IV

## 1 Introduction

Medial open-wedge high tibial osteotomy (OWHTO) is an established surgical procedure for medial compartment osteoarthritis and varus malalignment. Traditionally, its therapeutic mechanism has been understood as static load redistribution via mechanical axis shift, effectively unloading the medial compartment and improving symptoms 1, 2, 3. Increasing evidence suggests that varus malalignment contributes to stress-dependent joint opening and shear loading, resulting in dynamic instability. Therefore, correction of alignment by OWHTO may function as a mechanical stabilizer rather than merely a load-redistributing procedure.

Recent biomechanical data provide important insight into optimal alignment targets in medial meniscus posterior root tear (MMPRT).^4^ In a controlled cadaveric simulation study, Hiranaka et al. demonstrated that medial compartment contact pressure in MMPRT knees normalizes at 60–65% weight-bearing line (WBL) without repair and at 50–55% WBL with repair.^5^ In addition, bone morphology further influences instability risk. Okazaki et al. reported that a steep medial proximal tibial slope (PTS) and shallow medial tibial plateau depth significantly increase the risk of MMPRT.^6^ A steeper PTS increases anterior tibial translation under load, amplifying shear forces across the posterior root attachment.^7^ Thus, sagittal alignment contributes not only to load magnitude but also to dynamic instability.

OWHTO requires release of the superficial medial collateral ligament (sMCL), a primary valgus stabilizer. Although intraoperative valgus laxity increases after sMCL release, longitudinal data show that postoperative laxity does not persist after repair and fixation 8, 9, 10. Studies examining MCL release report that increased medial joint opening during OWHTO returns toward pre-release levels after plate fixation, and no significant postoperative valgus laxity is observed at mid-to long-term follow-up.^10^ This suggests that alignment correction and soft-tissue restoration can reestablish coronal stability despite temporary intraoperative laxity.

Despite these advances, the dynamic stabilizing effect of OWHTO under stress conditions has not been fully quantified. Most prior studies evaluated static radiographic parameters or contact pressure, rather than stress-induced instability. Furthermore, the relationship between structural alignment correction, dynamic stabilization, and clinical outcome remains unclear. The purpose of this study was therefore to evaluate dynamic coronal and sagittal stability using intraoperative fluoroscopic stress assessment and to determine how alignment correction influences postoperative stabilization. We hypothesized that OWHTO would reduce dynamic instability and that greater preoperative instability would predict greater stabilization.

## 2 Patients and methods

### 2.1 Patients

This study included 38 consecutive knees in 35 patients who underwent medial open-wedge high tibial osteotomy for symptomatic medial compartment pathology associated with varus alignment. Indications for OWHTO included medial compartment osteoarthritis and alignment-dependent instability. This study included 31 knees with MMPRT associated with coronal malalignment, defined as a mechanical axis percentage of ≤30% or a femorotibial angle (FTA) of ≥180°. Previous studies have shown that isolated meniscal repair in such varus-aligned knees results in inferior outcomes.^11^, ^12^ Concomitant MMPRT repair was performed in 10 knees using a pull-out technique.^13^ Patients with severe ligament insufficiency (>10° coronal laxity), advanced lateral compartment degeneration, inflammatory arthritis, flexion contracture >15°, or incomplete radiographic data were excluded. The patient demographics are shown in Table 1.

**Table 1:** Demographic and clinical characteristics.

		Range
Number of patients/knees	35/38	
Gender, male/female	13/22	
Age (years)	59.3 ± 8.9	44 − 75
Height (cm)	164 ± 10	143 − 179
Body weight (kg)	72.5 ± 15.9	48 − 105
Body mass index (kg/m^2^)	26.9 ± 4.3	18 − 34
Kellgren-Lawrence grade	2.6 ± 0.6	2 − 4
Hip-knee-ankle varus angle (°)	4.6 ± 2.4	1.0 - 8.2

The values are presented as mean ± standard deviation.

The hip-knee-ankle (HKA) angle represents whole-limb mechanical alignment measured using the mechanical axes of the femur and tibia, with varus alignment expressed as a positive value.

This retrospective study was approved by the Institutional Ethics Committee of Kagawa Rosai Hospital (approval No. R7-45, approval date: March 13, 2026). The study was conducted in accordance with the principles of the Declaration of Helsinki. Written informed consent for surgery and the use of clinical data for research purposes was obtained from all patients. This study involved retrospective analysis of anonymized clinical data obtained during routine clinical practice.

### 2.2 Surgical procedure

A longitudinal skin incision of approximately 6–8 cm was made over the anteromedial proximal tibia, starting distal to the joint line and extending toward the pes anserinus. The pes anserinus and sMCL were carefully dissected in a subperiosteal fashion to allow adequate gap opening while preserving tissue integrity. Under fluoroscopic control, a biplanar osteotomy was performed after insertion of guide pins. The primary horizontal cut was made oblique to the tibial plateau, approximately 3–4 cm distal to the medial joint line, extending toward the lateral hinge. A secondary ascending osteotomy was created proximally at the level of the tibial tuberosity. The osteotomy gap was gradually opened under fluoroscopic control using a mechanical spreader, with the limb positioned in hyperextension and the spreader preferentially inserted posteriorly to minimize an increase in PTS (Fig. 1a). Coronal correction was guided by preoperative planning to achieve a postoperative weight-bearing line at approximately 55–60% of the tibial plateau width using an alignment rod (Fig. 1b). To achieve slight reduction of PTS, the osteotomy gap was opened symmetrically in the anterior and posterior cortices. The osteotomy gap was filled with wedge-shaped artificial bone graft (Osferion 60, OSferion Biomaterials, Japan) to match the osteotomy geometry (Fig. 1c). The sMCL and periosteum were repaired to prevent postoperative valgus instability (Fig. 1d). After achieving the planned correction and restoring medial soft tissue tension, a medial locking plate system (TriS, OSferion Biomaterials, Japan) was applied to provide stable fixation (Fig. 1e and f).

**Fig. 1:**
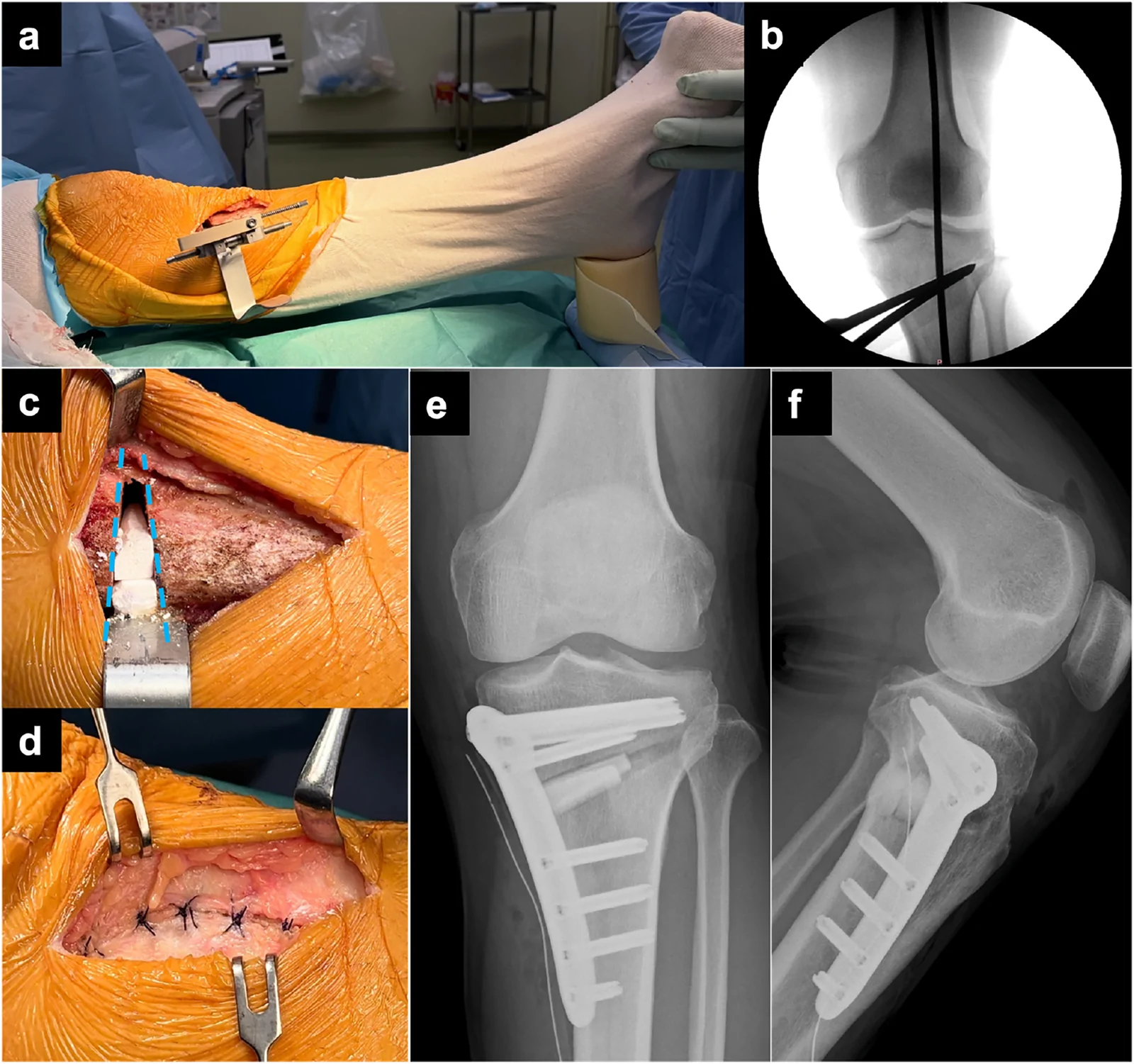
Key intraoperative steps of medial OWHTO. (a) Opening of the osteotomy gap with the lower limb maintained in extension and posterior placement of the spreader to minimize an unintended increase in PTS. (b) Coronal alignment confirmation using an alignment rod. (c) Insertion of wedge-shaped artificial bone graft; dashed line indicates a slight wedge configuration. (d) Repair of the superficial medial collateral ligament and periosteum. (e) Postoperative anteroposterior radiograph. (f) Postoperative lateral radiograph.

Postoperatively, range of motion was permitted immediately, with non-weight bearing for 1 week, progression to one-third partial weight bearing at week 1, and full weight bearing from week 2 onward.

### 2.3 Fluoroscopic assessment

#### 2.3.1 Coronal stability assessment

Intraoperative fluoroscopic evaluation was conducted to assess dynamic coronal stability before and after osteotomy correction. With the knee maintained in full extension and neutral rotation, standardized anteroposterior images were obtained while maximal manual varus and valgus stress was applied by a single experienced examiner (Fig. 2a and b). Coronal stability was quantified using two parameters: the joint line convergence angle (JLCA), representing intra-articular stability and defined as the angle between the distal femoral condylar tangent and the tibial plateau line, and the FTA, representing extra-articular coronal alignment and defined as the angle between the anatomical axes of the femur and tibia.^14^, ^15^ Dynamic coronal instability was expressed as the absolute difference between varus and valgus measurements for each parameter, defined as dynamic JLCA (dJLCA) and dynamic FTA (dFTA), respectively.

**Fig. 2:**
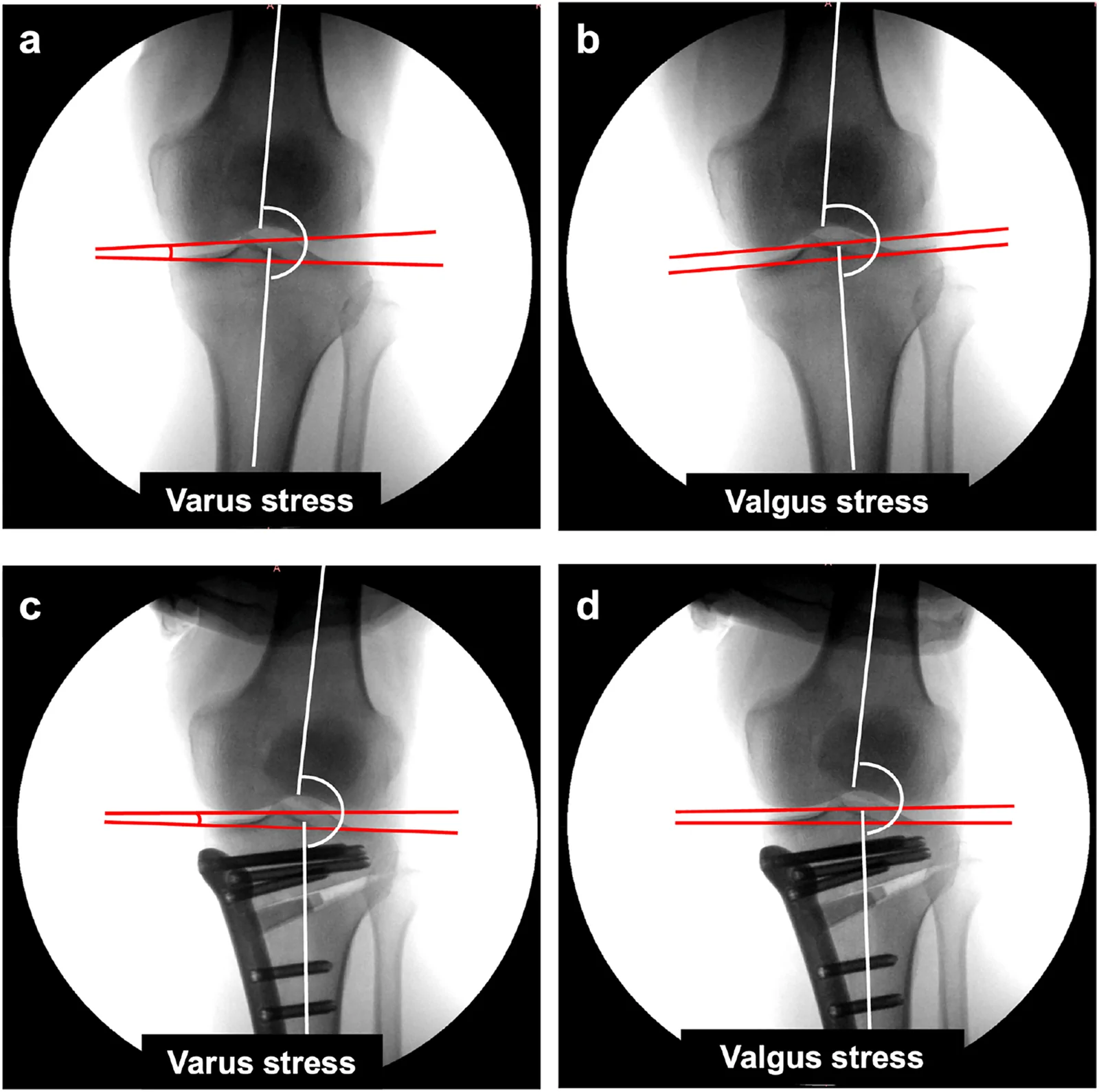
Intraoperative fluoroscopic assessment of coronal stability. (a) Preoperative varus stress view; white lines indicate FTA and red lines indicate JLCA. (b) Preoperative valgus stress view. (c) Postoperative varus stress view. (d) Postoperative valgus stress view. Comparison of stress views demonstrates reduced JLCA and FTA after osteotomy.

#### 2.3.2 Sagittal stability assessment

With the knee maintained in mid-flexion (approximately 60–80°), maximal manual anterior drawer stress was applied to the proximal tibia while stabilizing the femur (Fig. 3a and b). A true lateral image with superimposed femoral condyles was obtained to minimize rotational error. Anterior translation was defined as the perpendicular distance from the anterior tibial cortex to Blumensaat's line (ATB) as described.^16^ To minimize magnification bias, this distance was normalized to the anteroposterior tibial plateau length and expressed as a percentage. ATB was measured before osteotomy and reassessed after plate fixation. The change in ATB (ΔATB) represented the degree of dynamic sagittal stabilization. All stress examinations were performed by a single experienced surgeon using a consistent manual technique to minimize inter-examiner variability.

**Fig. 3:**
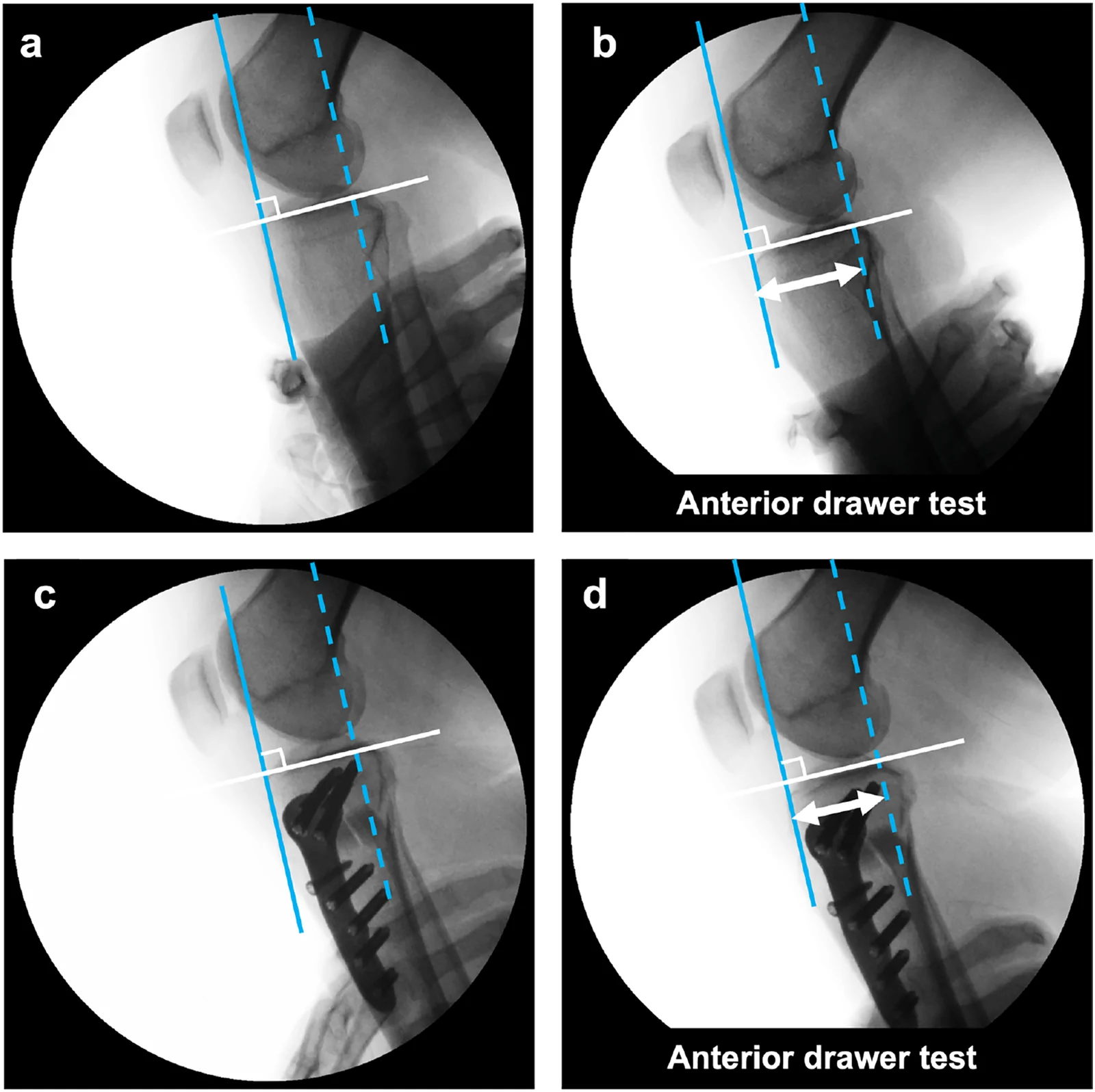
Intraoperative fluoroscopic assessment of sagittal stability. (a) Preoperative lateral view; a white line indicates the tibial plateau reference line, a blue line indicates the perpendicular line from the anterior tibial cortex, and the blue dashed line indicates the perpendicular line from the posterior point of the Blumensaat line. (b) Preoperative lateral view under anterior drawer stress; the arrow indicates anterior tibial translation to the Blumensaat line (ATB). (c) Postoperative lateral view. (d) Postoperative lateral view under anterior drawer stress.

### 2.4 Radiographic analysis

Standardized anteroposterior and lateral radiographs were obtained preoperatively and at final follow-up. Coronal alignment was evaluated using the medial proximal tibial angle (MPTA), defined as the medial angle between the tibial plateau and the anatomical axis of the tibia. Sagittal alignment was assessed using PTS, measured on lateral radiographs as the angle between the medial tibial plateau and a line perpendicular to the anatomical tibial axis as described.^17^ The changes in alignment parameters (ΔMPTA and ΔPTS) were correlated with dynamic stability parameters (dJLCA, dFTA, and ΔATB) to examine the association between alignment correction and dynamic stabilization.

### 2.5 Clinical evaluation

Clinical outcomes were evaluated preoperatively and at 6 and 12 months postoperatively using the Lysholm score, International Knee Documentation Committee (IKDC) subjective score, and the Knee injury and Osteoarthritis Outcome Score (KOOS), including its subscales (Symptoms, Pain, Activities of Daily Living, Sport/Recreation, and Quality of Life).

### 2.6 Statistical analysis

All statistical analyses were performed using SPSS software (IBM SPSS Statistics, version 25.0; IBM Corp., Armonk, NY, USA). Preoperative and postoperative comparisons were performed using the Wilcoxon signed-rank test. Statistical significance was set at p < 0.05. For the MMPRT subgroup analysis, continuous variables were compared between the concomitant repair and non-repair groups using the Mann–Whitney *U* test. Correlations between alignment parameters (ΔMPTA and ΔPTS) and dynamic stability parameters (dJLCA, dFTA, and ΔATB) were analyzed using Spearman's rank correlation coefficient. The strength of correlation was interpreted as weak (|ρ| < 0.30), moderate (0.30–0.60), or strong (>0.60). Interobserver reliability for dJLCA, dFTA, and ATB measurements was evaluated using the intraclass correlation coefficient (ICC) based on a two-way random-effects model with absolute agreement, by two experienced surgeons (T.M. and H.K.). ICC values were interpreted as follows: <0.50 poor, 0.50–0.75 moderate, 0.75–0.90 good, and >0.90 excellent reliability. A post hoc power analysis was conducted to estimate the adequacy of the sample size for detecting correlations between alignment correction and dynamic stability parameters. With a sample size of 31 knees, a two-tailed α level of 0.05, and an expected moderate effect size (r = 0.50), the calculated statistical power exceeded 0.80.

## 3 Results

### 3.1 Radiographic alignment and dynamic stability

Significant improvements in both coronal and sagittal alignment were achieved following osteotomy (Table 2). FTA and MPTA demonstrated significant correction toward valgus alignment, and PTS was significantly reduced postoperatively (all p < 0.001). Dynamic coronal instability parameters, including dJLCA and dFTA, significantly decreased after correction (p < 0.001), indicating improved coronal stability (Fig. 2c and d). Similarly, ATB significantly decreased postoperatively (p < 0.001), reflecting improved sagittal stability (Fig. 3c and d). Interobserver reliability was excellent for dJLCA (ICC = 0.91), dFTA (ICC = 0.88), and ATB (ICC = 0.93), indicating high reproducibility of measurements.

**Table 2:** Changes in radiographic alignment and stability parameters.

	Pre-operation	Post-operation	p value
FTA (°)	178.6 ± 1.7	171.9 ± 1.4	< 0.001
MPTA (°)	84.3 ± 1.9	91.1 ± 1.9	< 0.001
PTS (°)	8.5 ± 2.6	6.5 ± 2.2	< 0.001
dJLCA (°)	3.5 ± 1.6	1.5 ± 0.7	< 0.001
dFTA (°)	5.6 ± 2.6	2.2 ± 1.2	< 0.001
ATB (%)	16.2 ± 5.8	6.1 ± 3.5	< 0.001

*Significance was determined with use of the Wilcoxon signed-rank test. *p* < 0.05.

*FTA* Femoral tibial angle, *MPTA* Medial proximal tibial angle, *PTS* Posterior tibial slope, *dJLCA* Dynamic joint line convergence angle, *dFTA* Dynamic femoral tibial angle, *ATB* Anterior tibial translation to Blumensaat line.

In a subgroup analysis of knees with MMPRT, no significant differences were observed between the concomitant MMPRT repair and non-repair groups in preoperative ATB, postoperative ATB, and ΔATB (all p > 0.05). The magnitude of ΔATB was comparable between the repair and non-repair groups (10.6 ± 3.1% vs. 10.8 ± 8.2%, respectively; p = 0.489). Similarly, no significant difference was observed in ΔdJLCA between the two groups (p = 0.672).

### 3.2 Correlation between preoperative instability and postoperative improvement

Preoperative instability parameters were significantly correlated with the magnitude of postoperative stabilization (Table 3). Preoperative dJLCA and dFTA showed strong positive correlations with their respective postoperative improvements. Preoperative ATB demonstrated a strong negative correlation with ΔATB. Preoperative PTS was moderately correlated with ΔPTS, while MPTA showed a weaker association with its postoperative change. These findings indicate that greater preoperative instability and malalignment were associated with greater dynamic stabilization after osteotomy.

**Table 3:** Correlation between preoperative parameters and postoperative improvement.

Preoperative parameter	Change parameter	Spearman ρ	p value
dJLCA	ΔdJLCA	0.794	< 0.001
dFTA	ΔdFTA	0.765	< 0.001
MPTA	ΔMPTA	−0.382	< 0.001
PTS	ΔPTS	0.530	0.015
ATB	ΔATB	−0.765	< 0.001

*dJLCA* Dynamic joint line convergence angle, *dFTA* Dynamic femoral tibial angle, *MPTA* Medial proximal tibial angle, *PTS* Posterior tibial slope, *ATB* Anterior tibial translation to Blumensaat line.

### 3.3 Clinical outcomes

All clinical scores significantly improved at both 6 and 12 months postoperatively compared with preoperative values (all p < 0.001) (Table 4).

**Table 4:** Postoperative changes of clinical scores.

	Pre-operation	6 months	12 months	p value
Lysholm score	64.8 ± 11.5	89.4 ± 5.9	95.8 ± 4.2	< 0.001
IKDC score	36.6 ± 16.2	63.7 ± 15.9	75.9 ± 13.5	< 0.001
KOOS				
Pain	59.4 ± 19.4	83.9 ± 13.1	91.2 ± 9.1	< 0.001
Symptom	60.1 ± 21.8	79.0 ± 15.6	87.2 ± 11.0	< 0.001
ADL	68.8 ± 15.5	87.0 ± 8.9	93.3 ± 7.7	< 0.001
Sport/Rec	26.9 ± 23.2	51.8 ± 27.4	73.6 ± 20.4	< 0.001
QOL	32.3 ± 20.6	60.1 ± 17.4	74.6 ± 18.8	< 0.001

*IKDC* International Knee Documentation Committee subjective knee evaluation form, *KOOS* Knee Injury and Osteoarthritis Outcome Score, *ADL* activities of daily living, *Sport/Rec* sport and recreation function, *QOL* quality of life.

*Significance was determined with use of the Wilcoxon signed-rank test. *p* < 0.05.

P-values represent comparison between preoperative and 12-month postoperative values.

## 4 Discussion

This study demonstrates that OWHTO reduces stress-induced instability in both the coronal and sagittal planes, and that the magnitude of stabilization correlates with preoperative instability. These findings suggest that dynamic laxity in varus knees is primarily alignment-dependent and mechanically reversible. Accordingly, preoperative stress fluoroscopic parameters, including dJLCA, dFTA, and ATB, may serve as practical functional indicators of correctable instability.

The reduction in dJLCA and dFTA indicates restoration of medial soft-tissue balance. JLCA reflects load-responsive capsuloligamentous behavior rather than fixed osseous deformity, and has been shown to correlate with intra-articular deformity and soft-tissue laxity.^14^ Postoperative changes in JLCA after high tibial osteotomy also represent adjustments in medial soft-tissue tension, supporting its role as a dynamic parameter rather than a static osseous metric.^18^ The strong association between preoperative instability and postoperative improvement suggests that coronal laxity is largely attributable to malalignment rather than intrinsic ligament insufficiency. Although sMCL release is required during OWHTO, postoperative valgus laxity does not persist when medial structures are repaired and fixation is stable.^8^ The present findings support the concept that valgus correction restores effective medial ligament tension. In addition, OWHTO is performed proximal to the tibial tuberosity with an oblique biplanar cut, thereby preserving patellar tendon tension. Previous biomechanical and radiographic studies have demonstrated that increasing the opening gap may elevate patellofemoral contact pressure and alter patellar height.^19^ As the gap is preferentially opened in the posteromedial part, compressive loading within the tibiofemoral joint increases, influencing joint contact mechanics beyond simple coronal realignment.^1^, ^20^ Thus, these mechanical changes may contribute to enhanced coronal and sagittal stability after OWHTO.

The reduction in ATB indicates improved sagittal stability. PTS has been shown to influence anterior tibial translation and tibiofemoral shear forces, with increasing slope significantly elevating anterior translation and ligament loading.^21^, ^22^ Even small changes in slope can substantially alter anterior translation under load. Clinical series of OWHTO have demonstrated that controlled slope modification of approximately 2° is achievable and results in measurable reduction of anterior tibial translation in ACL-deficient knees.^23^ The observed association between ΔPTS and ΔATB supports the concept that sagittal alignment contributes to dynamic stabilization. Control of PTS during osteotomy therefore has mechanical relevance beyond radiographic correction. When slope reduction exceeding 2° is required particularly in ACL-deficient knees, anterior closing-wedge or lateral closed-wedge osteotomy may be necessary to achieve adequate sagittal correction and effective control of anterior tibial translation 24, 25, 26.

Many knees in the present cohort had MMPRT with varus malalignment, a combination that increases medial load and dynamic instability. MMPRT disrupts hoop function and significantly elevates medial compartment contact pressure, creating a meniscectomy-like biomechanical environment.^27^ Clinical studies have demonstrated that OWHTO improves symptoms in these knees and may reduce meniscal extrusion when varus alignment is corrected.^28^, ^29^ Furthermore, biomechanical evidence suggests that optimal alignment targets differ depending on root status, supporting individualized correction strategies rather than uniform neutral alignment.^5^ In our cohort, greater preoperative instability was associated with greater stabilization after correction, suggesting that the laxity in MMPRT-varus knees is largely attributable to malalignment. PTS may further influence root biomechanics. Increased PTS induces tibial translation and shear stress, whereas modest slope reduction during OWHTO may improve the loading environment. Whether concomitant MMPRT repair is necessary during OWHTO remains controversial. Previous studies have suggested that root repair combined with OWHTO may enhance meniscal healing and cartilage regeneration.^30^ In contrast, other studies have reported comparable clinical outcomes after OWHTO with or without concomitant root repair, suggesting that correction of varus malalignment itself may improve the biomechanical environment of the medial compartment.^31^, ^32^ In the present subgroup analysis, sagittal stabilization assessed by ΔATB did not significantly differ between the MMPRT repair and non-repair groups. Similarly, no significant difference was observed in coronal stabilization assessed by ΔdJLCA. These findings provide a biomechanical perspective on the ongoing debate regarding concomitant root repair. The improvement in dynamic stability even without MMPRT repair suggests that correction of osseous malalignment by OWHTO itself may improve both coronal and sagittal stability. This does not imply that MMPRT repair is unnecessary, because root repair may provide additional benefits for meniscal healing and cartilage preservation.^30^ Rather, our findings suggest that the dynamic stabilizing effect of OWHTO may be substantially driven by osseous alignment correction, while meniscal repair may provide additional benefits by restoring meniscal function. Further studies are needed to determine which patients benefit from concomitant MMPRT repair.

All clinical scores improved significantly at 6 and 12 months. Mechanical instability under load has been implicated in pain and functional limitation in knees with malalignment and osteoarthritis.^33^ By reducing stress-induced joint excursion, OWHTO may attenuate abnormal shear forces and enhance perceived stability. Improvements in alignment and knee stability have been associated with better clinical outcomes and patient-reported functional scores after medial opening-wedge HTO.^34^ Accordingly, alignment correction should be viewed not only as load redistribution but also as dynamic stabilization, which may represent a key mechanism underlying clinical improvement in varus-aligned knees.

This study has several limitations. First, its retrospective design and relatively small sample size may limit generalizability, although post hoc power analysis suggested adequate power for detecting moderate correlations. Second, stress fluoroscopic assessment was performed manually by a single experienced examiner using a consistent technique to minimize variability; however, the magnitude of the applied stress was not objectively quantified. Therefore, some variability in the applied force cannot be excluded. Future studies using a dynamometer or mechanized stress device may provide more standardized assessment of dynamic knee stability. In addition, the knee flexion angle was maintained within approximately 60–80° but was not objectively standardized to a fixed angle. Because knee flexion angle influences cruciate ligament tension and anterior tibial translation, differences in flexion angle between pre- and post-osteotomy measurements may have affected ATB values. Third, although significant clinical improvement was observed at 12 months, this follow-up period is insufficient to determine whether the observed dynamic stabilization contributes to long-term joint preservation. Mid-to long-term follow-up, including evaluation at 3 to 5 years, will be necessary to determine the clinical relevance of these findings. Finally, the majority of included knees had MMPRT, which may limit extrapolation to other pathologies. However, this distribution reflects the common clinical indication for OWHTO in varus-aligned knees.

These findings suggest that alignment correction through OWHTO may provide functional stabilization in addition to load redistribution. In particular, preoperative stress parameters, including dJLCA, dFTA, and ATB, provide an objective assessment of dynamic instability and may assist in selecting adjunctive procedures such as meniscal repair or slope modification.

## 5 Conclusions

This study demonstrates that OWHTO not only corrects alignment but also acts as a dynamic stabilizer by restoring medial collateral ligament tension and preserving patellar tendon mechanics. Stress fluoroscopic evaluation may serve as a practical tool for identifying alignment-driven instability and guiding surgical planning.

## Author contributions

Y.O. collected and analyzed the data and wrote the first draft of the manuscript. T.M. and H.K. assessed radiographic parameters and reviewed the manuscript. K.K. and N.C. reviewed the manuscript. All authors read and approved the final version of the manuscript.
